# Study on the adsorption performance of coal gangue-loaded nano-FeOOH for the removal of Pb^2+^, Cu^2+^, and Cd^2+^ from acid mine drainage

**DOI:** 10.1039/d5ra03306c

**Published:** 2025-06-30

**Authors:** Xuying Guo, Xiaoyue Zhang, Zilong Zhao, Yanrong Dong, Honglei Fu, Fanbo Meng, Wei Sun

**Affiliations:** a College of Science, Liaoning Technical University Fuxin 123000 Liaoning China guoxuying@lntu.edu.cn +86-24-13941834560; b College of Civil Engineering, Liaoning Technical University Fuxin 123000 Liaoning China; c College of Mining, Liaoning Technical University Fuxin 123000 Liaoning China; d College of Environmental Science and Engineering, Liaoning Technical University Fuxin 123000 Liaoning China

## Abstract

In view of the serious pollution caused by heavy metals such as Pb^2+^, Cu^2+^ and Cd^2+^ present in acid mine drainage (AMD) and the difficulty in their treatment, based on the stable mineral skeleton structure of coal gangue and the good adsorption of nano-FeOOH, coal gangue-loaded nano-FeOOH (nFeOOH-CG) was successfully prepared *via* chemical precipitation. The effects of nFeOOH-CG on the removal of Pb^2+^, Cu^2+^ and Cd^2+^ from AMD were systematically investigated under different adsorbent dosage, initial pH, reaction time and initial concentration conditions. The adsorption mechanism of nFeOOH-CG was revealed using the adsorption isotherm, adsorption kinetics, adsorption thermodynamic model, SEM-EDS, XRD, TEM, FTIR spectroscopy and BET characterization. Results indicated that the rate of Pb^2+^, Cu^2+^ and Cd^2+^ removal by nFeOOH-CG reached 96.85%, 88.38% and 73.1%, respectively, under the conditions of 5 g per L dosage; pH 4; 150 min reaction time and 100 mg per L initial concentration of Pb^2+^, Cu^2+^ and Cd^2+^, which were significantly better than those of unmodified coal gangue. The process of Pb^2+^, Cu^2+^ and Cd^2+^ adsorption by nFeOOH-CG conforms to the Langmuir and pseudo-second-order kinetic models, indicating that the adsorption mechanism mainly involves monolayer and chemical adsorption. The adsorption of Pb^2+^ and Cu^2+^ by nFeOOH-CG is a spontaneous, endothermic and entropy-increasing process, while the adsorption of Cd^2+^ is a non-spontaneous, endothermic and entropy-increasing process. Characterization analysis showed that the specific surface area of nFeOOH-CG was 103.68 m^2^ g^−1^, which was 13.22 times higher than that of coal gangue. The loading of nFeOOH significantly increased the specific surface area and surface active sites of coal gangue. The adsorption mechanism of nFeOOH-CG on Pb^2+^, Cu^2+^ and Cd^2+^ was mainly attributed to the increased specific surface area, surface complexation, electrostatic attraction and ion exchange. This study provides a theoretical basis and technical reference for the efficient resource utilization of coal gangue and the environmental remediation of AMD.

## Abstract 2

Based on the stable mineral skeleton structure of coal gangue and the good adsorption of nano-FeOOH, coal gangue-loaded nano-FeOOH (nFeOOH-CG) was successfully prepared *via* chemical precipitation.

## Graphical abstract

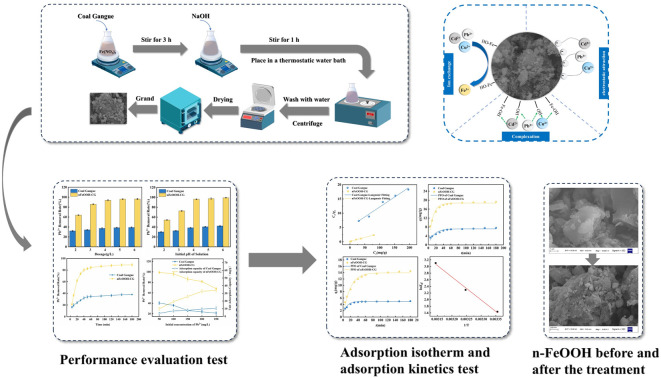

## 1 Introduction

China is one of the world's leading producers of coal. While coal mining has significantly contributed to the country's socio-economic development and helped meet the increasing demand for mineral resources, it generates substantial quantities of acid mine drainage (AMD).^1–3^ As AMD is rich in heavy metal ions, it easily causes heavy metal pollution, which seriously endangers the ecological environment and human health.^4–6^ Fe^2+^ and Mn^2+^ ions dominate the AMD system with their high concentrations,^7,8^ but such pollutant ions can usually be effectively controlled using conventional precipitation methods.^9^ In contrast, although Pb^2+^, Cu^2+^ and Cd^2+^ are often present at low concentrations, they have become target pollutants that need priority treatment because of their strong chemical toxicity and irreversible ecotoxicological effects. At present, AMD treatment technologies mainly include chemical precipitation,^10^ ion exchange,^11^ biological^12^ and adsorption^13^ methods. Among them, the adsorption method has attracted much attention owing to its advantages of simple operation, controllable cost and strong adaptability. Adsorption materials are the key to the application of the adsorption method. However, among the adsorbents currently used, such as ion exchange resin,^14^ biochar,^15^ activated carbon^16^ and graphene,^17^ ion exchange resin encounter the problem of high regeneration cost. Although commercial activated carbon and graphene have high adsorption capacity, their preparation process is complicated, mainly involving high-temperature activation or chemical vapor deposition. However, cheap biochar generally has defects such as poor selectivity and slow adsorption rate. This technical and economic contradiction has prompted us to develop new adsorption materials based on industrial solid waste. By combining solid waste coal gangue with nano-FeOOH, we can not only inherit the cost advantage of biochar, but also use the specific coordination ability of FeOOH to achieve efficient adsorption of heavy metal ions.

Coal gangue, the solid waste produced in the process of coal mining and washing, has become a key problem restricting the sustainable development of coal energy industry.^18–20^ At present, coal gangue is mainly used in filling materials, production of building materials, power generation and so on.^21^ Most of these disposal methods have low added value, and high-added-value application technology is relatively scarce. Therefore, it has become an urgent problem to explore the economic, efficient and green resource utilization of coal gangue. In recent years, a large number of researchers have been committed to using coal gangue as an adsorbent in the field of environmental pollution remediation^22,23^ based on the inorganic components such as silicon and aluminum and the porous structure characteristics of coal gangue. Fan^24^ explored the repair effect of fresh coal gangue on mine water through dynamic tests. The results showed that the removal rate of Pb^2+^ by fresh coal gangue reached 57.28%, but the removal rates of Cu^2+^ and Cd^2+^ were only 27.38% and 42.67%. It can be seen that when untreated coal gangue is directly used as an adsorbent, the adsorption capacity is not strong and the adsorption activity is low, which limits its practical application efficiency. Gao *et al.*^25^ used coal gangue as a raw material to prepare ZSM-5 molecular sieve, and the theoretical maximum adsorption capacity of Pb^2+^ and Cu^2+^ could reach 232.56 mg g^−1^ and 118.34 mg g^−1^, respectively. Thermal activation can improve the adsorption capacity of heavy metals, but there is a risk of secondary pollution. Shang *et al.*^26^ prepared sulfhydryl modified coal gangue by modifying coal gangue with trimethoxysilane. The results showed that the maximum adsorption capacities of Pb^2+^, Cu^2+^ and Hg^2+^ were 332.8 mg g^−1^, 110.4 mg g^−1^ and 179.2 mg g^−1^, respectively. Surfactant modification can improve selectivity, but it requires expensive reagents. These traditional modification methods have the disadvantages of high processing cost, complex process and instability, and it is difficult to achieve industrial promotion. Ma *et al.*^27^ used 5% acetic acid solution to dissolve chitosan and controlled the mass ratio of chitosan/modified coal gangue to be 0.09. The coal gangue/chitosan composite material was prepared by impregnation method, and the composite material particles were obtained. Under the optimum conditions, the removal rate of Cr^6+^ reached 97.57%. Wang *et al.*^28^ prepared coal gangue-loaded Fe/FeO_*x*_ by a chemical liquid-phase reduction method. Under the optimal conditions, the removal rate of Cd^2+^ reached 99.12%. It can be seen that loading other substances on coal gangue will effectively increase its adsorption performance.

Iron-based oxides are widely used as adsorbents in the field of environmental remediation due to their excellent redox activity, high specific surface area and environmental friendliness. Among them, nano-FeOOH exhibits strong adsorption and catalytic degradation ability for heavy metal ions due to its high specific surface area and abundant hydroxyl groups on the surface. Mohamed *et al.*^29^ prepared nano-FeOOH by a chemical precipitation method and studied the adsorption of Pb^2+^. The results indicated that the theoretical maximum adsorption capacity of nano-FeOOH for Pb^2+^ can reach 15.11 mg g^−1^. However, the mechanical strength of FeOOH nanoparticles is weak and they are easy to aggregate, which limits their large-scale application. To this end, researchers have tried to load nano-FeOOH on porous substrate materials to enhance their dispersibility and mechanical strength. Hao *et al.*^30^ prepared mesoporous alumina–carbon nanosheet-loaded nano-FeOOH particles by hydrothermal synthesis to remove Cr^6+^ in wastewater, and the adsorption capacity of Cr^6+^ was increased to 120 mg g^−1^. Li *et al.*^31^ studied the performance of Mn/FeOOH composites as adsorbents in the removal of Pb^2+^ and Cd^2+^. Under optimal conditions, the removal capacity of Pb^2+^ and Cd^2+^ was 18% and 40% higher than that of FeOOH, respectively. It can be seen that the selection of carrier materials with a stable structure to load nano-FeOOH for preparing composite adsorption materials can effectively overcome the shortcomings of nano-materials such as easy agglomeration and weak mechanical strength and greatly improve the adsorption performance. The coal gangue has a stable structure and contains multiple clays, which can be used as a mineral skeleton to provide an ideal loading platform for nanomaterials. Therefore, coal gangue was used as a mineral carrier to load nano-FeOOH to construct a composite material for AMD remediation.

Based on this, this study used mineral loading technology and chemical precipitation method to prepare a composite adsorption material, coal gangue-loaded nano-FeOOH (nFeOOH-CG), with cost advantages and high adsorption performance, and applied it to the treatment of Pb^2+^, Cu^2+^ and Cd^2+^ in AMD. The effects of adsorbent dosage, initial pH, contact time and initial concentration of solution on the adsorption of Pb^2+^, Cu^2+^ and Cd^2+^ by nFeOOH-CG were investigated. The adsorption mechanism of Pb^2+^, Cu^2+^ and Cd^2+^ on nFeOOH-CG was revealed by adsorption isotherm, adsorption kinetics, adsorption thermodynamics, SEM-EDS, XRD, FTIR spectroscopy and BET. It provides technical reference for the high value utilization of coal gangue and the repair method of acid mine wastewater.

## 2 Materials and methods

### 2.1. Experimental materials

Coal gangue samples were collected from Fuxin City, Liaoning Province (42.01°N, 121.65°E). The material was sieved to 90–120 mesh, washed three times with deionized water, and dried at 378.15 K in a forced-air drying oven (Model DHG-9030, Shanghai Yiheng Scientific Instruments Co., Ltd, China) prior to experimentation. The main chemical components of coal gangue were determined by X-ray fluorescence spectrometry (XRF), and the results are shown in Table 1.

**Table 1:** Main compositions of Fuxin coal gangue

Component	SiO_2_	TiO_2_	Al_2_O_3_	Fe_2_O_3_	MnO	MgO	CaO	Na_2_O	K_2_O	P_2_O_5_	SO_3_
Content (%)	57.63	0.80	14.60	9.15	0.22	5.41	7.91	1.03	2.34	0.27	0.64

The following analytical-grade reagents were employed: NaOH, HNO_3_, Fe(NO_3_)_3_·9H_2_O, Pb(NO_3_)_2_, Cu(NO_3_)_2_·3H_2_O, and Cd(NO_3_)_2_·4H_2_O, all obtained from Sinopharm Chemical Reagent Co., Ltd (Shanghai, China). Deionized water was used throughout the experimental procedures.

Simulated AMD wastewater: the simulated heavy metal ion concentration was formulated with reference to the actual water quality of a coal mining area in Shanxi Province. Pb(NO_3_)_2_, Cu(NO_3_)_2_·3H_2_O and Cd(NO_3_)_2_·4H_2_O were dissolved in deionized water to obtain a simulated water sample containing Pb^2+^, Cu^2+^ and Cd^2+^ at a concentration of 100 mg L^−1^. The simulated AMD wastewater was treated by simulated AMD wastewater. The pH of the solution was adjusted to 4 with 0.1 mol per L HNO_3_ and 0.1 mol per L NaOH.

### 2.2. Experimental methods

#### 2.2.1. Preparation of nFeOOH-CG

**Fig. 1:**
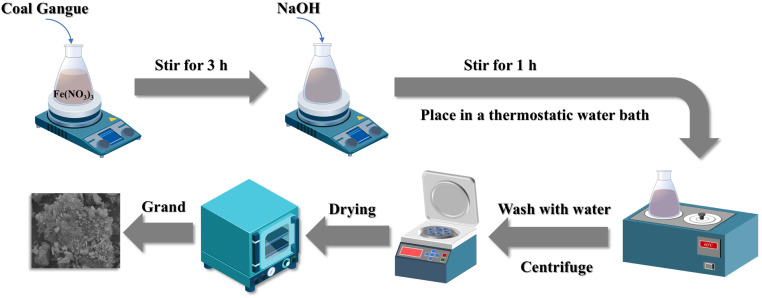
Preparation process for nFeOOH-CG.

Pretreated coal gangue (10 g) with a particle size of 90–120 mesh was put into 200 mL of 0.25 mol per L Fe(NO_3_)_3_ solution and stirred at 300 rpm for 3 h using a magnetic stirrer (Suzhou Guohua Instrument Co., 84-1, Ltd, China). The suspension was rapidly titrated with 1 mol per L NaOH under continuous stirring until the reaction system reached a pH of 12. The mixture was then transferred to a constant-temperature oscillator (Model SHA-8, Jiangsu Guowang Instrument Co., Ltd, China) and maintained at 60 °C for 60 h. The resulting product was filtered, washed to neutral pH, dried at 80 °C in an electric blast drying oven (Model DHG-9030A, Shanghai Yiheng Scientific Instruments Co., Ltd, China), and ground to obtain the nFeOOH-CG (Fig. 1).

#### 2.2.2. Performance evaluation methods

To assess the potential of nFeOOH-CG as an adsorbent, its performance in removing Pb^2+^, Cu^2+^, and Cd^2+^ from AMD was systematically evaluated. Batch experiments investigated the effects of adsorbent dosage (2–6 g L^−1^), initial pH (2–6), contact time (5–180 min), and initial metal concentration (50–250 mg L^−1^) on the removal rate. A control group containing equivalent masses of raw coal gangue (90–120 mesh) was tested for comparison. Triplicate trials were conducted for all conditions, with mean values reported. The removal rate (*η*, %) and adsorption capacity (*q*_e_, mg g^−1^) were calculated using the following equations:1
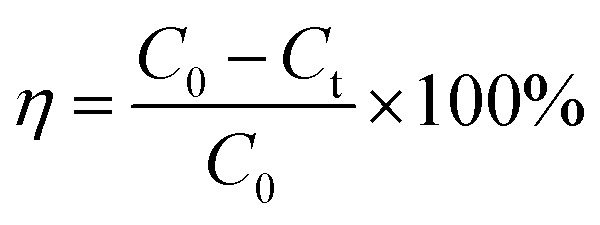
2
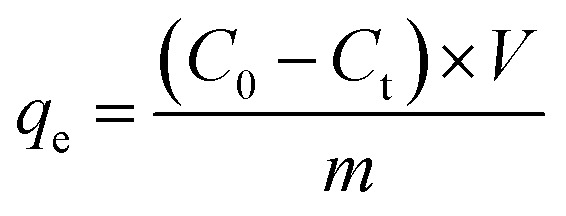
where: *η* is the removal rate, %; *C*_o_ and *C*_t_ represent the initial and residual concentrations of the target ions, respectively, mg L^−1^; *q*_e_ is the adsorption capacity, mg g^−1^; *V* is the solution volume, *L*; *m* is the adsorbent mass, *g*.

##### 2.2.2.1 Experimental steps

Under the conditions of room temperature of 298.15 K, initial solution pH of 4, and initial concentrations of Pb^2+^, Cu^2+^, and Cd^2+^ of 100 mg L^−1^, nFeOOH-CG with different masses (2, 3, 4, 5, and 6 g L^−1^) was weighed into 200 mL simulated wastewater, and a magnetic stirrer was used to stir at 150 rpm for 150 min. The concentration was measured to investigate the effect of adsorbent dosage on the removal of Pb^2+^, Cu^2+^, and Cd^2+^ in AMD by nFeOOH-CG. Under the conditions of room temperature of 298.15 K, initial pH values of the solution of 2, 3, 4, 5, and 6, initial concentration of Pb^2+^, Cu^2+^, and Cd^2+^ of 100 mg L^−1^, nFeOOH-CG with a mass of 5 g L^−1^ was weighed and put into 5 groups of 200 mL simulated wastewater. A magnetic stirrer was used to stir for 150 min at a speed of 150 rpm. Finally, the sample was analyzed, and its concentration was measured to investigate the effect of initial pH of the solution on the removal of Pb^2+^, Cu^2+^, and Cd^2+^ in AMD by nFeOOH-CG. Under the conditions of room temperature of 298.15 K, initial solution pH of 4, initial concentration of Pb^2+^, Cu^2+^, and Cd^2+^ of 100 mg L^−1^, nFeOOH-CG with a mass of 5 g L^−1^ was weighed into 200 mL simulated wastewater, and a magnetic stirrer was used to stir at a speed of 150 rpm for 5, 10, 20, 30, 40, 50, 60, 90, 120, 150 and 180 min. After sampling and detecting its concentration, the effect of contact time on the removal of Pb^2+^, Cu^2+^, and Cd^2+^ in AMD by nFeOOH-CG was investigated. Under the conditions of room temperature of 298.15 K, initial pH of 4, initial concentrations of Pb^2+^, Cu^2+^, and Cd^2+^ of 50, 100, 150, 200, 250 mg L^−1^, nFeOOH-CG with a mass of 5 g L^−1^ was added to 5 groups of 200 mL simulated wastewater. A magnetic stirrer was used to stir at a speed of 150 rpm for 150 min. After sampling and detection of its concentration, the effect of nFeOOH-CG on the removal of Pb^2+^, Cu^2+^, and Cd^2+^ in AMD was investigated.

#### 2.2.3. Adsorption isotherm, kinetics, and thermodynamics experiments

##### 2.2.3.1 Pb^2+^, Cu^2+^, and Cd^2+^ adsorption isotherm experiment in AMD

At 298.15 K, the initial pH of the solution was 4, and the initial concentrations of Pb^2+^, Cu^2+^, and Cd^2+^ were 50, 100, 150, 200, and 250 mg L^−1^, respectively. nFeOOH-CG with a mass of 5 g L^−1^ was added to 200 mL of simulated wastewater, and a magnetic stirrer was used to stir at a speed of 150 rpm. When the reaction was carried out for 150 min, the residual concentration of Pb^2+^, Cu^2+^, and Cd^2+^ was determined after passing through a 0.45 μm filter membrane. Each group of experiments were repeated three times, and the mean value was taken.

The adsorption of Pb^2+^, Cu^2+^, and Cd^2+^ on coal gangue and nFeOOH-CG was isothermally fitted by Langmuir and Freundlich adsorption isotherm equations to better evaluate the adsorption reaction mechanism. The equation is as follows:3
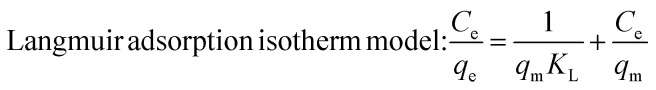

4 Freundlich adsorption isotherm model: *q*_e_ = *K*_F_*C*_e_^1/*n*^

5
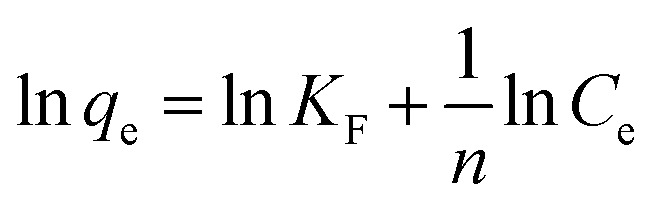
where: *C*_e_ is the equilibrium concentration of the pollutant, mg L^−1^; *q*_e_ is the equilibrium adsorption capacity, mg g^−1^; *q*_m_ is the maximum monolayer adsorption capacity, mg g^−1^; *K*_L_ is the Langmuir constant; *K*_F_ is the Freundlich constant; *n* is the adsorption intensity factor.

##### 2.2.3.2 Adsorption kinetics of Pb^2+^, Cu^2+^, and Cd^2+^ in AMD

Under the conditions of room temperature of 298.15 K, initial pH of 4, initial concentration of Pb^2+^, Cu^2+^, and Cd^2+^ of 100 mg L^−1^, nFeOOH-CG with a mass of 5 g L^−1^ was added to 200 mL simulated wastewater and stirred at 150 rpm using a magnetic stirrer. After stirring for 5, 10, 20, 30, 40, 50, 60, 90, 120, 150 and 180 min, the remaining Pb^2+^, Cu^2+^, and Cd^2+^ concentrations in the solution were determined. Each group of experiments were repeated three times, and the mean value was taken.

Lagergren first-order adsorption kinetic model, Lagergren second-order adsorption kinetic model and intraparticle diffusion model were used to fit Pb^2+^, Cu^2+^, and Cd^2+^ in coal gangue and nFeOOH-CG-treated AMD. The linear expression of the adsorption kinetic model is as follows:

6 Lagergren first-order adsorption kinetic model: ln(*q*_e_ − *q*_*t*_) = ln *q*_e_ − *k*_1_*t*

7 *q*_*t*_ = *q*_e_(1 − e^−*k*_1_*t*^)

8
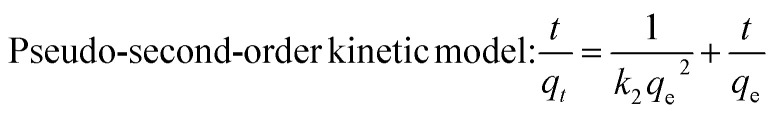

9 Intra-particle diffusion model: *q*_*t*_ = *k*_p_*t*^1/2^ + *C*

where *q*_*t*_ is the adsorption capacity at time, mg g^−1^; *q*_e_ is the equilibrium adsorption capacity, mg g^−1^; *k*_1_ is the pseudo-first-order rate constant, min^−1^; *k*_2_ is the pseudo-second-order rate constant, mg g^−1^ min^−1^; *k*_p_ is the intra-particle diffusion rate constant, mg g^−1^ min^−1/2^.

##### 2.2.3.3 Adsorption thermodynamics of Pb^2+^, Cu^2+^, and Cd^2+^ in AMD

Under the conditions of room temperature of 298.15 K, 308.15 K and 318.15 K, initial pH of 4 and initial concentration of Pb^2+^, Cu^2+^, and Cd^2+^ of 100 mg L^−1^, nFeOOH-CG with a mass of 5 g L^−1^ was added to three groups of 200 mL simulated wastewater and stirred at a speed of 150 rpm using a magnetic stirrer. When the reaction was carried out for 150 min, the residual concentration of Pb^2+^, Cu^2+^, and Cd^2+^ was determined after passing through a 0.45 μm filter membrane. Each group of experiments were repeated three times, and the mean value was taken.

Through the experimental data of Pb^2+^, Cu^2+^, and Cd^2+^ adsorption by nFeOOH-CG, the effect of temperature on the adsorption effect was discussed and the thermodynamic characteristics of adsorption were analyzed. The equation is as follows:10
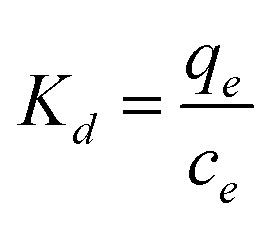

11 Δ*G* = −*RT* ln *K*

12 Δ*G* = Δ*H* − *T*Δ*S*

13
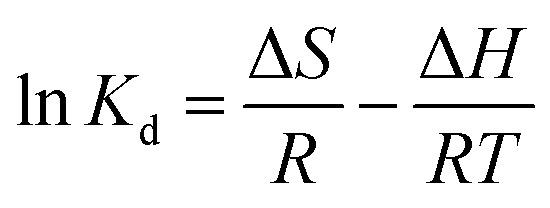
where: Δ*G* is the Gibbs free energy change, kJ mol^−1^; Δ*H* is the enthalpy change, kJ mol^−1^; Δ*S* is the entropy change, J mol^−1^ K^−1^; *K*_d_ is the thermodynamic equilibrium constant; *R* is the gas constant, 8.314 J mol^−1^ K^−1^; *T* is the temperature, K.

#### 2.2.4. Leaching toxicity test method

According to the “solid waste leaching toxicity method-sulfuric acid/nitric acid method” (HJ/T300-2007), sulfuric acid and nitric acid with a mass ratio of 2/1 were added to deionized water to make the solution pH 3.1–3.3. The dried coal gangue and nFeOOH-CG were added to the above extract at a solid–liquid ratio of 1/10 (g mL^−1^) and stirred at room temperature for 18 h. The concentration of main heavy metal ions was detected using a flame atomic spectrophotometer. Three parallel samples were set up in each group, and the average value was taken as the result.

#### 2.2.5. Regeneration test of nFeOOH-CG

The reusability of nFeOOH-CG was evaluated by three cycles of adsorption–desorption experiments. In the experiment, 200 mL of simulated AMD wastewater was taken each time, and 5 g per L nFeOOH-CG adsorbent was added. The adsorption process was completed by stirring at 150 rpm for 150 minutes at room temperature. Subsequently, 0.5 mol per L EDTA eluent was used for desorption and regeneration treatment, followed by stirring for 3 h. After desorption, the adsorbent was centrifuged, cleaned and dried to be reused for Pb^2+^, Cu^2+^ and Cd^2+^ adsorption experiments under the same experimental conditions. Three parallel samples were set up in each group, and the average value was taken as the result.

#### 2.2.6. Water quality analysis and material characterization methods

Pb^2+^, Cu^2+^, and Cd^2+^ were determined using a flame atomic spectrophotometer (GB/7475-87, China) at 283.3, 324.7 and 228.8 nm wavelengths, and the pH was determined by a glass electrode method (GB/T 6920-86).

The surface morphology of the materials was analyzed by scanning electron microscopy coupled with energy-dispersive spectroscopy (SEM-EDS, SIGMA 500, Carl Zeiss AG, Germany). Measurements were conducted at 10 μA current and 15 kV accelerating voltage, under argon protection, with magnification ranging from 5000× to 100 00×. X-ray diffraction (XRD, D8 ADVANCE, Bruker Corporation, Germany) was employed for mineralogical composition analysis and crystal structure determination. The measurements used a Cu-Kα radiation source (40 kV, 30 mA) with a scanning range of 2*θ* = 10°–90°, a step size of 0.02°, and a scanning speed of 0.5 s per step. A Fourier transform infrared (FTIR) spectrometer (VERTEX 70, Bruker Corporation, Germany) was used to identify surface functional groups. The samples were dried, ground to <0.074 mm, mixed with KBr at 1/100 ratio, and pressed into pellets. Spectra were recorded in the 400–4000 cm^−1^ wavenumber range. The specific surface area and pore size distribution were determined by the Brunauer–Emmett–Teller (BET) method using a Micromeritics ASAP 2460 analyzer (Micromeritics Instrument Ltd, USA). The samples were degassed at 200 °C for 6 h under vacuum prior to N_2_ adsorption measurements.

## 3 Results and discussion

### 3.1. Performance evaluation experiments

#### 3.1.1. Effect of adsorbent dosage

**Fig. 2:**
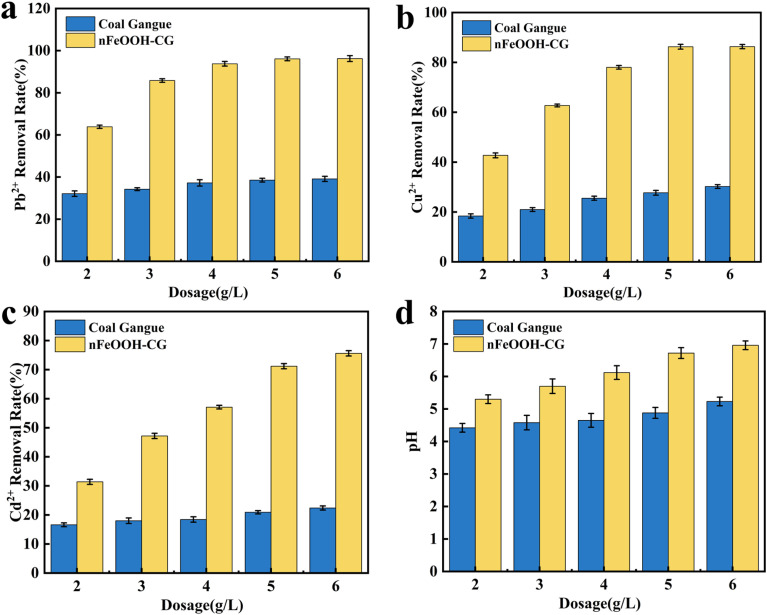
Effect of adsorbent dosage on the removal of Pb^2+^, Cu^2+^, and Cd^2+^. (a) Effect of adsorbent dosage on the Pb^2+^ removal rate. (b) Effect of adsorbent dosage on the Cu^2+^ removal rate. (c) Effect of adsorbent dosage on the Cd^2+^ removal rate. (d) Effect of adsorbent dosage on pH. (Experimental conditions: initial pH = 4; contact time = 150 min; initial concentration of Pb^2+^, Cu^2+^, and Cd^2+^ = 100 mg L^−1^; temperature = 298.15 K; stirring speed = 150 rpm).

It can be seen from Fig. 2 that with the increase in nFeOOH-CG dosage from 2.0 g L^−1^ to 5.0 g L^−1^, the removal rates of Pb^2+^, Cu^2+^ and Cd^2+^ increased significantly to 96.25%, 86.34% and 75.62%, respectively, and the pH value of the solution also increased. When the dosage was more than 5.0 g L^−1^, the removal behavior of the three metal ions was different: the removal efficiency of Pb^2+^ and Cu^2+^ increased by less than 2%, while the removal rate of Cd^2+^ still increased slightly from 75.62% to 78.15%. This is because with the increase in nFeOOH-CG dosage, the specific surface area of the composite increases, and the effective adsorption sites of Pb^2+^, Cu^2+^, and Cd^2+^ increase, thereby adsorbing and removing a large amount of pollutants.^32^ However, when the dosage increases to a certain extent, the adsorption site of the composite material is excessive, but the residual pollutants in the solution are less, resulting in a small increase in the removal rate, and the utilization rate of the composite material is greatly reduced.^33^ In addition, at a large dosage, agglomeration occurs between the adsorbents, resulting in the stacking of adsorption sites, resulting in a decrease in the specific surface area, limiting the adsorption capacity of the material.^34^ In summary, under the premise of ensuring high pollutant removal rate and saving adsorbent dosage, 5 g L^−1^ was selected as the adsorbent dosage for subsequent experiments.

#### 3.1.2. Effect of initial pH

**Fig. 3:**
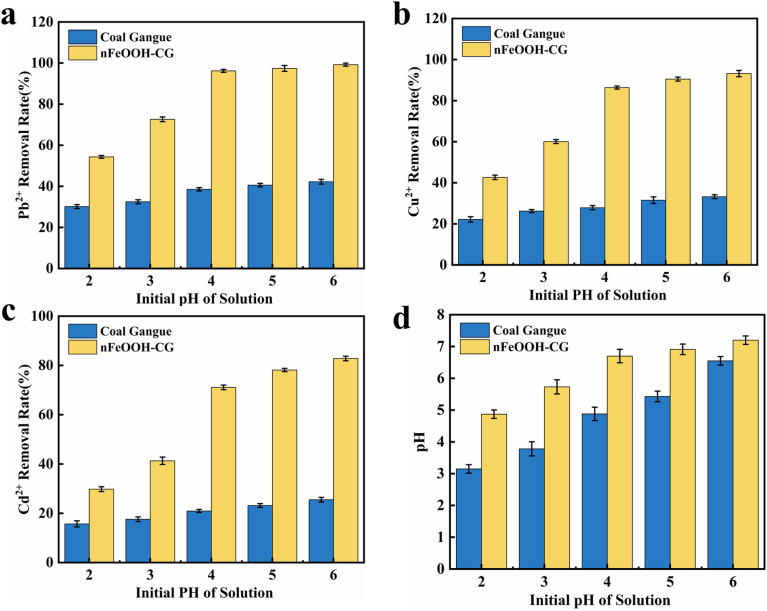
Effect of initial pH on the removal of Pb^2+^, Cu^2+^, and Cd^2+^. (a) Effect of initial pH on the Pb^2+^ removal rate. (b) Effect of initial pH on the Cu^2+^ removal rate. (c) Effect of initial pH on the Cd^2+^ removal rate. (d) Effect of initial pH on solution pH. (Experimental conditions: adsorbent dosage = 5 g L^−1^; contact time = 150 min; initial concentration of Pb^2+^, Cu^2+^, and Cd^2+^ = 100 mg L^−1^; temperature = 298.15 K; stirring speed = 150 rpm).

It can be seen from Fig. 3 that with the increase in the initial pH of the solution, the removal rate and pH of Pb^2+^, Cu^2+^, and Cd^2+^ in AMD by nFeOOH-CG increased rapidly and then stabilized, and the effect was significantly better than that of coal gangue. When the initial pH value of the solution increased from 2 to 4, the removal rates of Pb^2+^, Cu^2+^, and Cd^2+^ by nFeOOH-CG gradually increased. When the initial pH value of the solution was 4, the removal rates of Pb^2+^, Cu^2+^, and Cd^2+^ reached 96.21%, 86.38% and 71.1%, respectively, and the pH of the solution reached 6.7 after the reaction. When the initial pH value of the solution increased from 4 to 6, the removal rate of Cd^2+^ by nFeOOH-CG still increased slightly, while the removal rate of Pb^2+^ and Cu^2+^ tended to be stable. This is because the initial pH of the solution will affect the electrostatic interaction between the pollutants and the surface of the adsorbent material, and the physical and chemical adsorption reactions such as complexation reaction and ion exchange.^35^ In the lower pH range, H^+^ in the solution occupies a large number of adsorption sites on the surface of nFeOOH-CG and competes with Pb^2+^, Cu^2+^, and Cd^2+^ to adsorb.^36^ At the same time, the surface hydroxyl groups of nano-FeOOH loaded under acidic conditions were protonated to form ≡FeOH^2+^, which was mutually exclusive with Pb^2+^, Cu^2+^, and Cd^2+^, resulting in a lower removal rate of Pb^2+^, Cu^2+^, and Cd^2+^ by nFeOOH-CG under lower pH conditions (pH = 2–3).^37^ With the increase in pH, the concentration of H^+^ in the solution system decreased, which was beneficial to reduce the competition between H^+^ and Pb^2+^, Cu^2+^, and Cd^2+^, so that more adsorption sites remained on the surface of nFeOOH-CG, which was used to adsorb and remove the target pollutants. The high concentration of OH^−^ in the solution will increase the negative charge on the surface of nFeOOH-CG and reduce the electrostatic repulsion with Pb^2+^, Cu^2+^, and Cd^2+^, which is beneficial to the adsorption or formation of insoluble precipitate and improvement of the removal effect. In summary, nFeOOH-CG can maintain high adsorption performance under different pH conditions, showing a wide range of application potential for AMD treatment. Considering the pH of the actual wastewater and the experimental results, the pH value of 4 was selected for subsequent experiments.

#### 3.1.3. Effect of reaction time on the removal of Pb^2+^, Cu^2+^, and Cd^2+^

**Fig. 4:**
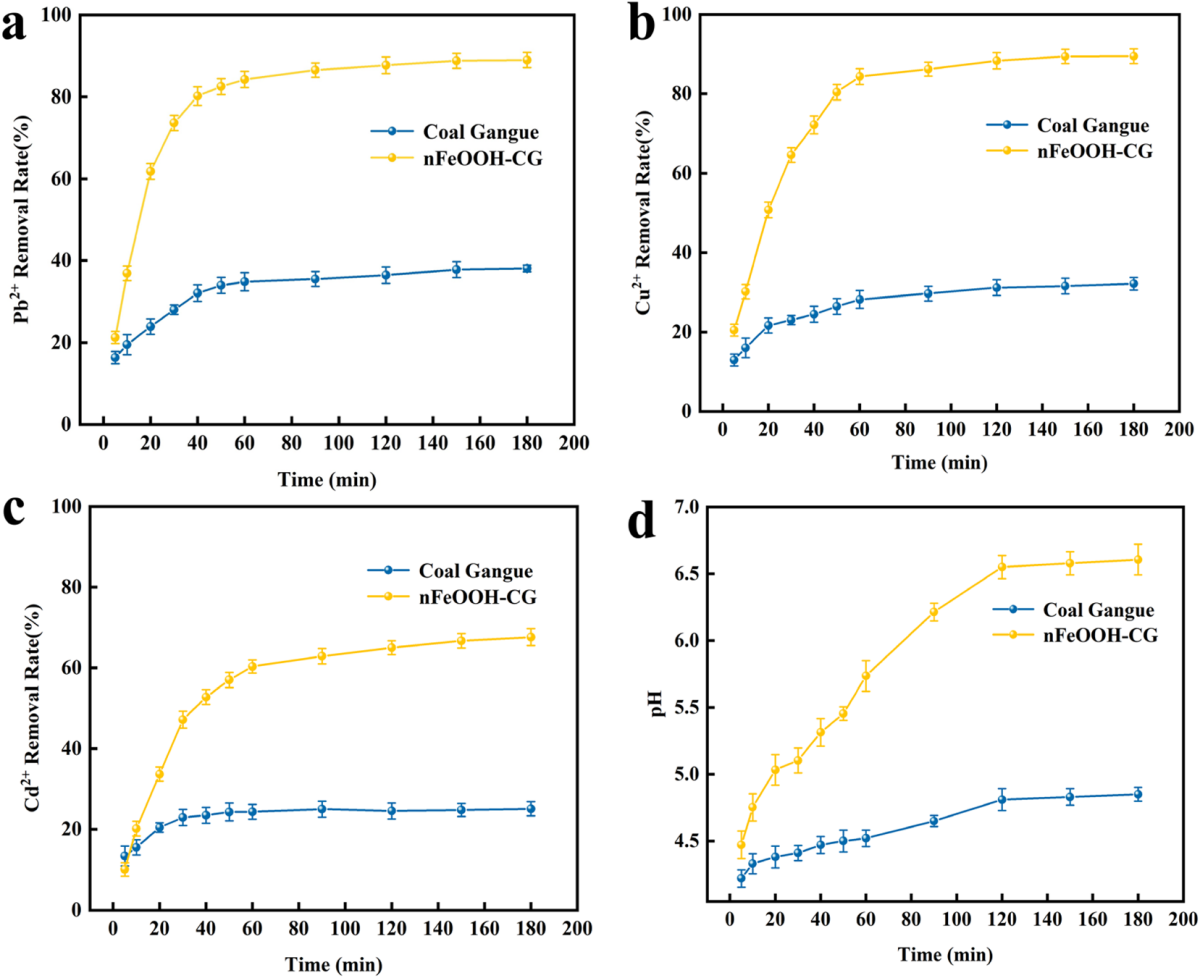
Effect of reaction time on the removal of Pb^2+^, Cu^2+^, and Cd^2+^. (a) Effect of reaction time on the Pb^2+^ removal rate. (b) Effect of reaction time on the Cu^2+^ removal rate. (c) Effect of reaction time on the Cd^2+^ removal rate. (d) Effect of reaction time on solution pH. (Experimental conditions: adsorbent dosage = 5 g L^−1^; initial pH = 4; initial concentration of Pb^2+^, Cu^2+^, Cd^2+^ = 100 mg L^−1^; temperature = 298.15 K; stirring speed = 150 rpm).

It can be seen from Fig. 4 that the removal rate and pH of Pb^2+^, Cu^2+^, and Cd^2+^ by nFeOOH-CG increased slowly with the increase in time, and the effect was obviously better than that of coal gangue. When the reaction time increased from 5 min to 150 min, the removal rate of Pb^2+^, Cu^2+^, and Cd^2+^ by nFeOOH-CG gradually increased. When the reaction time was 150 min, Pb^2+^, Cu^2+^, and Cd^2+^ reached 96.04%, 85.64% and 71.1%, respectively, and the pH of the solution reached 6.7 after the reaction. The removal rate of Cu^2+^ and Cd^2+^ by nFeOOH-CG still increased slightly from 150 to 200 min, while the removal rate of Pb^2+^ tended to be stable. This is because in the early stage of the reaction, the adsorption sites on the surface of nFeOOH-CG are sufficient, which can quickly bind to Pb^2+^, Cu^2+^, and Cd^2+^ in the solution, and then fix.^38^ At the same time, in the early stage of the reaction, the concentration of Pb^2+^, Cu^2+^, and Cd^2+^ in the solution is large. Under the concentration difference between the adsorbent material and the adsorbate, a certain mass transfer driving force is generated, which promotes the rapid adsorption of heavy metal ions to the surface of nFeOOH-CG. Therefore, the adsorption efficiency is high and the adsorption speed is fast in the early stage of the reaction. When the reaction time was 60–120 min, the number of adsorption sites on the surface of nFeOOH-CG was greatly reduced, the concentration of metal ions in the solution was also significantly reduced, and the mass transfer driving force was also weakened, so the adsorption rate was reduced.^39^ When the reaction time was more than 150 min, the removal rate of the three metal ions did not change significantly with time, indicating that the adsorption equilibrium state was basically reached at this time. In summary, in order to ensure that the adsorption equilibrium is reached, the subsequent experimental reaction time is set to 150 min.

#### 3.1.4. Effect of initial concentration

**Fig. 5:**
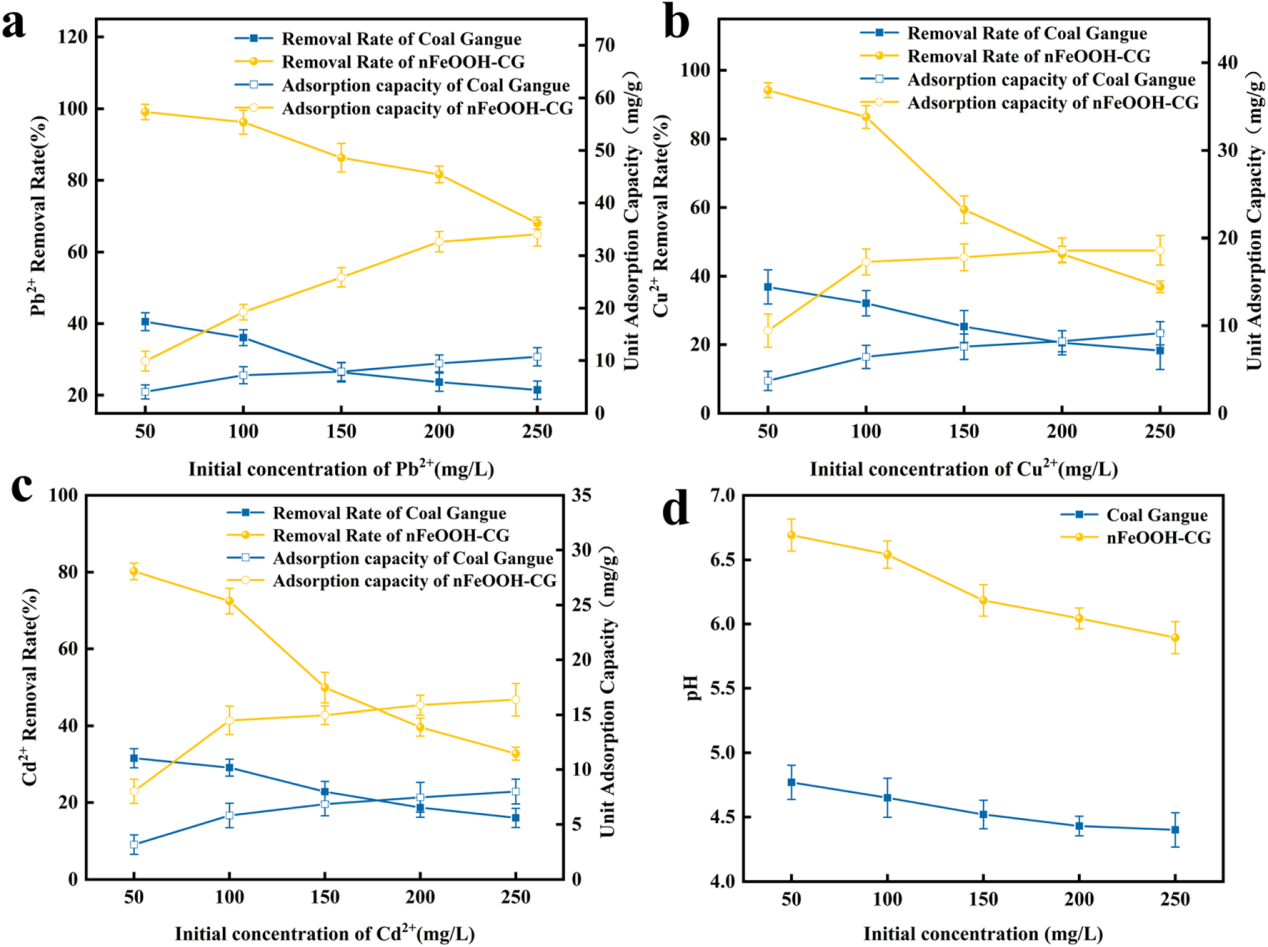
Effect of initial concentration on the removal of Pb^2+^, Cu^2+^, and Cd^2+^. (a) Effect of initial concentration on the Pb^2+^ removal rate. (b) Effect of initial concentration on the Cu^2+^ removal rate. (c) Effect of initial concentration on the Cd^2+^ removal rate. (d) Effect of initial concentration on solution pH. (Experimental conditions: adsorbent dosage = 5 g L^−1^, contact time = 150 min, initial pH = 4, temperature = 298.15 K, stirring speed = 150 rpm).

Fig. 5 shows the effect of different initial concentrations on the removal of Pb^2+^, Cu^2+^, and Cd^2+^. When the initial concentration of the solution increased from 50 to 100 mg L^−1^, the removal rate of Pb^2+^, Cu^2+^, and Cd^2+^ by nFeOOH-CG decreased gradually, and the adsorption capacity increased gradually. When the initial concentration of the solution was 100 mg L^−1^, the removal rates of Pb^2+^, Cu^2+^, and Cd^2+^ reached 96.15%, 94.3% and 80.23%, respectively. The adsorption capacities of Pb^2+^, Cu^2+^, and Cd^2+^ were 32.66 mg g^−1^, 18.59 mg g^−1^ and 15.87 mg g^−1^, respectively. When the concentration exceeded 100 mg L^−1^, the removal rate of Pb^2+^, Cu^2+^, and Cd^2+^ decreased rapidly, and the growth rate of adsorption capacity slowed down. The adsorption capacity of Cu^2+^ and Cd^2+^ in the range of 200–250 mg L^−1^ increased by only 3.2% and 2.5%, respectively. This is because Pb^2+^, Cu^2+^, and Cd^2+^ in the solution can fully react with nFeOOH-CG at low initial concentrations. As the initial concentration of the solution increases, the adsorption sites of nFeOOH-CG are not enough to provide sufficient reaction sites, and the removal rate decreases. However, in the high concentration environment, the interaction between Pb^2+^, Cu^2+^, and Cd^2+^ and the nFeOOH-CG binding site is also enhanced, which effectively reduces the mass transfer resistance between the solid/liquid phase, thereby improving the adsorption capacity, which also makes the adsorption capacity higher at high concentrations of heavy metal solutions.^40,41^ In summary, in order to ensure that the removal of Pb^2+^, Cu^2+^, and Cd^2+^ reached the ideal effect, 100 mg L^−1^ was selected as the initial concentration of subsequent experiments.

### 3.2. Analysis of adsorption isotherm results

**Fig. 6:**
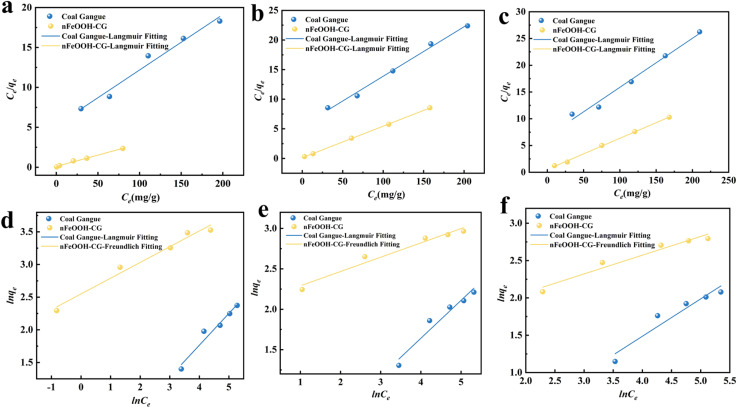
Adsorption isotherm model fitting curves for Pb^2+^, Cu^2+^, and Cd^2+^ by coal gangue and nFeOOH-CG. (a) Langmuir fitting for Pb^2+^ adsorption. (b) Langmuir fitting for Cu^2+^ adsorption. (c) Langmuir fitting for Cd^2+^ adsorption. (d) Freundlich fitting for Pb^2+^ adsorption. (e) Freundlich fitting for Cu^2+^ adsorption (f) Freundlich fitting for Cd^2+^ adsorption. (Experimental conditions: adsorbent dosage = 5 g L^−1^, initial pH = 4, contact time = 150 min, temperature = 298.15 K, stirring speed = 150 rpm).

Adsorption isotherms characterize equilibrium relationships between adsorbents and adsorbates at solid–liquid interfaces. The Langmuir and Freundlich models were applied to analyze nFeOOH-CG's adsorption behavior, with fitting results presented in Fig. 6 and Tables 2 and 3.

**Table 2:** Adsorption isotherm parameters for Pb^2+^, Cu^2+^ and Cd^2+^ adsorption by nFeOOH-CG

Isotherm model	Parameters	nFeOOH-CG		
		Pb^2+^	Cu^2+^	Cd^2+^
Langmuir model parameters	*q*_m_	35.1617	18.7652	17.1673
	*K*_L_	0.29241	0.46202	0.11004
	*R* ^2^	0.99312	0.99967	0.99797
Freundlich model parameters	*n*	4.14800	5.64493	3.98628
	*K*_F_	12.79238	8.27112	4.80088
	*R* ^2^	0.97036	0.95309	0.94168

**Table 3:** Adsorption isotherm parameters for Pb^2+^, Cu^2+^ and Cd^2+^ adsorption by coal gangue

Isotherm model	Parameters	Coal gangue		
		Pb^2+^	Cu^2+^	Cd^2+^
Langmuir model parameters	*q*_m_	14.4092	11.9603	10.9194
	*K*_L_	0.01325	0.01517	0.01363
	*R* ^2^	0.96691	0.99106	0.98154
Freundlich model parameters	*n*	2.05284	2.13188	1.99788
	*K*_F_	0.83117	0.79063	0.59690
	*R* ^2^	0.94092	0.93268	0.89998

According to the fitting curve, the experimental results show that the equilibrium adsorption capacity *q*_e_ of nFeOOH-CG increases with the increase of initial concentration of Pb^2+^, Cu^2+^, and Cd^2+^. The results showed that under high-concentration conditions, the interaction between heavy metal ions and adsorbents was more frequent, which promoted the adsorption process. By comparing the fitting effects of the two models, the Langmuir model is more suitable for describing the adsorption behavior of Pb^2+^, Cu^2+^, and Cd^2+^ by nFeOOH-CG, indicating that its adsorption characteristics are more inclined to monolayer adsorption. The adsorption sites on the surface of nFeOOH-CG are evenly distributed and the energy is equivalent. This also indicates that there are a large number of well-dispersed nano-FeOOH on the surface of nFeOOH-CG, and the high surface activity of nanocrystals improves the efficient and uniform adsorption characteristics of the composites. The theoretical maximum adsorption capacity of Pb^2+^, Cu^2+^, and Cd^2+^ by nFeOOH-CG was 35.16 mg g^−1^, 18.77 mg g^−1^ and 17.17 mg g^−1^, respectively, which was significantly improved compared with coal gangue. The correlation coefficient *n* of adsorption strength is greater than 1, indicating that the adsorption process is spontaneous. The value of *K*_F_ is proportional to the adsorption capacity and adsorption strength. The *K*_F_ values of coal gangue before loading are 0.8312, 0.7906 and 0.5969, respectively, and the *K*_F_ values after loading are 12.7924, 8.2711 and 4.8009, respectively. Therefore, the new coal gangue composites prepared by loading nano-FeOOH significantly improved the ability to treat Pb^2+^, Cu^2+^, and Cd^2+^ in AMD.

### 3.3. Analysis of adsorption kinetics

**Fig. 7:**
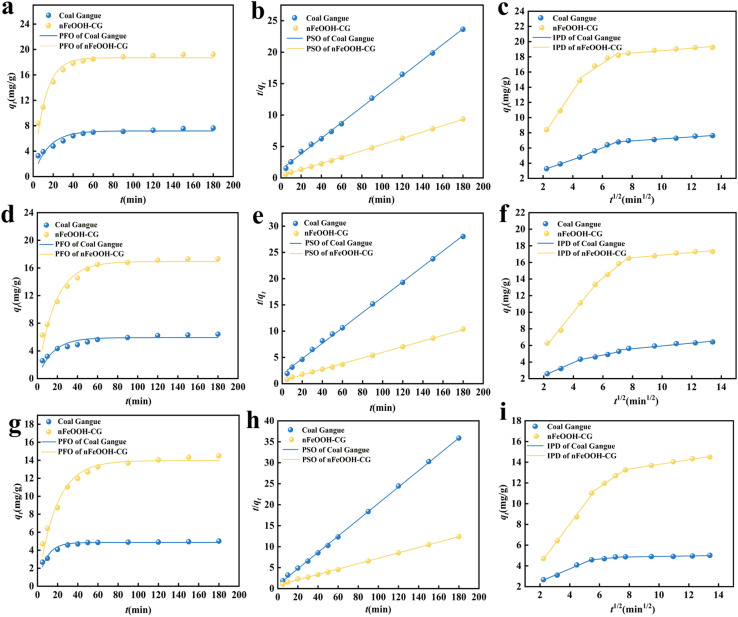
Kinetic model fittings for Pb^2+^, Cu^2+^, and Cd^2+^ adsorption by coal gangue and nFeOOH-CG. (a) Pb^2+^ pseudo-first-order kinetics. (b) Pb^2+^ pseudo-second-order kinetics. (c) Pb^2+^ intra-particle diffusion. (d) Cu^2+^ pseudo-first-order kinetics. (e) Cu^2+^ pseudo-second-order kinetics. (f) Cu^2+^ intra-particle diffusion. (g) Cd^2+^ pseudo-first-order kinetics. (h) Cd^2+^ pseudo-second-order kinetics. (i) Cd^2+^ intra-particle diffusion. (Experimental conditions: adsorbent dosage = 5 g L^−1^, initial pH = 4, initial concentration = 100 mg L^−1^, temperature = 298.15 K, agitation speed = 150 rpm).

The adsorption kinetics was analyzed using pseudo-first-order (PFO), pseudo-second-order (PSO), and intra-particle diffusion (IPD) models to identify rate-limiting mechanisms. The fitting results in Fig. 7 and Tables 4 and 5 demonstrate temporal adsorption behavior.

**Table 4:** Adsorption kinetics parameters for Pb^2+^, Cu^2+^and Cd^2+^ adsorption by nFeOOH-CG

Kinetic model	Parameters	nFeOOH-CG		
		Pb^2+^	Cu^2+^	Cd^2+^
Pseudo-first-order kinetic model	*k*_1_	0.0902	0.05806	0.05454
	*q*_e_	18.69665	16.93443	13.96059
	*R* ^2^	0.96268	0.95937	0.96718
Pseudo-second-order kinetic model	*k*_2_	8.0545 × 10^−3^	4.9509 × 10^−3^	5.0876 × 10^−3^
	*q*_e_	20.0160	18.5736	15.5618
	*R* ^2^	0.99962	0.99871	0.99934
Intra-particle diffusion model	*k*_p1_	2.91175	2.22806	1.92427
	*R* ^2^	0.99791	0.99275	0.99519
	*C*_1_	1.81788	1.09111	0.3362
	*k*_p2_	1.28965	1.42992	0.98387
	*R* ^2^	0.91793	0.98418	0.99359
	*C*_2_	9.40002	5.54729	5.66814
	*k*_p3_	0.16135	0.15538	0.22313
	*R* ^2^	0.9216	0.93125	0.9892
	*C*_3_	17.19336	15.33213	11.55963

**Table 5:** Adsorption kinetics parameters for Pb^2+^, Cu^2+^ and Cd^2+^ adsorption by coal gangue

Kinetic model	Parameters	Coal gangue		
		Pb^2+^	Cu^2+^	Cd^2+^
Pseudo-first-order kinetic model	*k*_1_	0.06748	0.06678	0.11586
	*q*_e_	7.17559	5.92294	4.8628
	*R* ^2^	0.86759	0.84482	0.91475
Pseudo-second-order kinetic model	*k*_2_	1.1678 × 10^−2^	1.1800 × 10^−2^	4.4906 × 10^−2^
	*q*_e_	8.04052	6.82221	5.13557
	*R* ^2^	0.99886	0.99864	0.99948
Intra-particle diffusion model	*k*_p1_	0.67954	0.78533	0.61334
	*R* ^2^	0.99998	0.99045	0.98742
	*C*_1_	1.7502	0.79081	1.25665
	*k*_p2_	0.79054	0.36271	0.13586
	*R* ^2^	0.98431	0.96722	0.89625
	*C*_2_	1.28623	2.66563	3.84538
	*k*_p3_	0.12973	0.16873	0.02491
	*R* ^2^	0.97307	0.91989	0.84913
	*C*_3_	5.90113	4.25575	4.6601

By comparing the *R*^2^ value of the kinetic model, it can be seen that the adsorption of Pb^2+^, Cu^2+^, and Cd^2+^ in AMD by nFeOOH-CG is more in line with the second-order kinetic fitting model. This indicates that the adsorption process is dominated by chemical adsorption. The *k*_1_ value of coal gangue is relatively low, which is related to its limited specific surface area and the number of active sites. The *k*_1_ value of nFeOOH-CG is relatively high, indicating that they can adsorb heavy metal ions faster. The modification of nFeOOH-CG increases the adsorption rate of heavy metal ions by increasing the surface active sites. The *k*_2_ value of nFeOOH-CG is higher, indicating that more chemical reaction steps are involved in the adsorption process. This further proves that the interaction between heavy metal ions and the surface of the adsorbent is not only physical adsorption, but also chemical adsorption. The fitting curve of the intraparticle diffusion model has no origin and the *k*_p_ value of nFeOOH-CG is higher, indicating that the contribution of internal diffusion to the adsorption rate is greater. The adsorption of Pb^2+^, Cu^2+^, and Cd^2+^ in AMD by nFeOOH-CG is controlled by two adsorption mechanisms: surface diffusion and pore diffusion. It can be seen from Fig. 7 that the fitting curve of nFeOOH-CG to the intraparticle diffusion model of Pb^2+^, Cu^2+^, and Cd^2+^ is a multi-stage fitting, indicating that the adsorption process can be divided into three stages: direct diffusion, intraparticle diffusion and dynamic equilibrium. In the first stage, due to the sufficient adsorption sites on the surface of nFeOOH-CG at the initial stage of the reaction, *q*_*t*_ changes rapidly with time, which is a rapid adsorption process. Pb^2+^, Cu^2+^, and Cd^2+^ diffused from the solution to the outer surface of nFeOOH-CG, and the diffusion mainly occurred in the micro-voids. In the second stage, the effective adsorption sites on the surface of nFeOOH-CG decreased, and the change rate of *q*_*t*_ decreased with time. After Pb^2+^, Cu^2+^, and Cd^2+^ adsorbed on the surface of nFeOOH-CG, they gradually diffused into the pores of nFeOOH-CG and filled the pores. In the third stage, the adsorption sites on the surface of nFeOOH-CG are close to saturation, and *q*_*t*_ almost does not change with time. In this stage, the diffusion rate decreases and gradually reaches equilibrium. The fitting curves did not pass through the origin, indicating that the adsorption of Pb^2+^, Cu^2+^, and Cd^2+^ by nFeOOH-CG was determined by both surface diffusion and intraparticle diffusion.

### 3.4. Analysis of adsorption thermodynamics

**Fig. 8:**
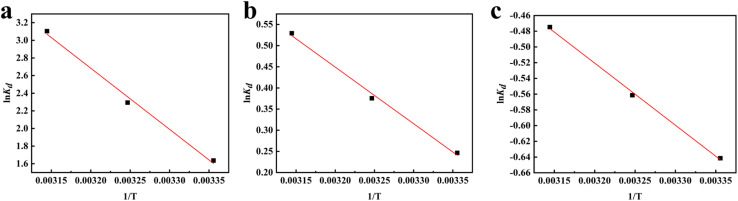
Thermodynamic model fittings for Pb^2+^, Cu^2+^, and Cd^2+^ adsorption by coal gangue and nFeOOH-CG. (a) Pb^2+^ adsorption thermodynamic fitting plot. (b) Cu^2+^ adsorption thermodynamic fitting plot. (c) Cd^2+^ adsorption thermodynamic fitting plot. (Experimental conditions: adsorbent dosage = 5 g L^−1^, initial pH = 4, initial concentration = 100 mg L^−1^, agitation speed = 150 rpm).

The adsorption thermodynamic behavior of nFeOOH-CG on heavy metal ions was studied at room temperatures of 298.15 K, 308.15 K, and 318.15 K, initial pH of 4, and initial concentration of Pb^2+^, Cu^2+^, and Cd^2+^ of 100 mg L^−1^, respectively. The thermodynamic change scatter distribution trend diagram was obtained with ln *K*_d_ as the longitudinal axis and 1/*T* as the horizontal axis, and it was linearly fitted as Fig. 8. The slope and intercept of the fitted line can be used to calculate the enthalpy change (Δ*H*) and entropy change (Δ*S*), and the results are shown in Tables 6 and 7.

**Table 6:** Adsorption thermodynamic parameters of nFeOOH-CG for the adsorption of Pb^2+^, Cu^2+^, and Cd^2+^

	Δ*G* (J mol^−1^)			Δ*H* (kJ mol^−1^)	Δ*S* (J mol^−1^ K^−1^)
	298 K	308 K	318 K		
Pb^2+^	−4.0527	−5.8708	−8.2047	57.7267	207.0436
Cu^2+^	−0.6102	−0.9614	−1.3991	11.1288	39.3476
Cd^2+^	1.5895	1.4376	1.2552	6.5648	16.6797

**Table 7:** Comparison of the properties of nFeOOH-CG and other adsorbents

Absorbent	*q*_m_ (mg g^−1^)			pH	Dosage (g L^−1^)	Reference
	Pb^2+^	Cu^2+^	Cd^2+^			
nFeOOH-CG	35.16	18.77	17.17	4	5	This study
Mg and Fe-LDHs AC		2.03		5	2.5	42
Algae silver nanoparticles	23.98		22.47	5	0.5	43
Vanadium doped titania	26			10	0.1	44
Walnut Husk extract-silver nanoparticles	7.38			6	2.0	45
Mechanochemical modified CG		5.69	6.73	6	1.67	46

It can be seen from Table 6 that the Gibbs free energy Δ*G* of Pb^2+^ and Cu^2+^ adsorbed by nFeOOH-CG at different temperatures is less than 0, indicating that the adsorption process can proceed spontaneously. With the increase in temperature, the absolute value of Δ*G* also increases, indicating that the increase of temperature is beneficial to the adsorption process. However, in the adsorption process of nFeOOH-CG on Cd^2+^, Δ*G* is positive, which means that the adsorption process of nFeOOH-CG on Cd^2+^ is thermodynamically non-spontaneous at these three test temperatures. From a thermodynamic point of view, it is not conducive to the natural occurrence of the adsorption process, which may be closely related to the hydration energy difference and surface complexation mechanism of the three metal ions. Although the Δ*G* value of Cd^2+^ adsorption is positive, its Δ*H* and Δ*S* are positive, indicating that the process needs to overcome the energy barrier, but the Fe–O–Cd bond formed after adsorption and the entropy increase caused by ion dehydration still drive the reaction. In contrast, the lower desolvation energy barriers of Pb^2+^ and Cu^2+^ make it easier to spontaneously complex with the hydroxyl groups on the surface of FeOOH. The Δ*H* values of Pb^2+^, Cu^2+^, and Cd^2+^ adsorbed by nFeOOH-CG are 57.7267 kJ mol^−1^, 11.1288 kJ mol^−1^ and 6.5648 kJ mol^−1^, respectively, indicating that the adsorption of Pb^2+^, Cu^2+^, and Cd^2+^ by nFeOOH-CG is an endothermic process, and the increase in temperature is beneficial to the reaction. The entropy changes Δ*S* were 207.0436 J mol^−1^ K^−1^, 39.3476 J mol^−1^ K^−1^, and 16.6797 J mol^−1^ K^−1^, respectively, which were all positive values, indicating that the randomness of the whole adsorption system increased during the reaction. It can be seen from Table 7 that nFeOOH-CG has better adsorption capacity for Pb^2+^, Cu^2+^, and Cd^2+^ than the other adsorbents reported in the literature.

### 3.5. Leaching toxicity analysis

The safety of adsorption materials in water treatment was evaluated by exploring the leaching toxicity of coal gangue and nFeOOH-CG. The content of heavy metals in leachate was determined using a flame atomic spectrophotometer with reference to Chinese national standards GB 5085.3-2007 and HJ/T299 2007. The contents of main heavy metals including Cr, Pb, Cu, Cd, Zn and Ni in coal gangue and nFeOOH-CG leaching solutions are shown in Table 8. It can be seen from the table that the leaching concentration of nFeOOH-CG is lower than the limit standard value of leaching toxicity (GB 5085.3-2007) and much lower than the leaching concentration of coal gangue, indicating that nFeOOH-CG has excellent immobilization ability for heavy metal ions. Under the condition of acidic pH = 4, –OH, Fe–OH and Fe–O on the surface of nFeOOH-CG share their non-bonded electron pairs with Pb^2+^, Cu^2+^ and Cd^2+^ and replace H in the group to form Fe–O–Pb, Fe–O–Cu and Fe–O–Cd surface complexes, which improve its safety in water treatment process, and nFeOOH-CG can be used as an excellent adsorbent for the treatment of wastewater containing Pb^2+^, Cu^2+^ and Cd^2+^.

**Table 8:** Contents of main heavy metals in coal gangue and nFeOOH-CG leachate (mg L^−1^)

Element	Mean value		Leaching toxicity standard value
	Coal gangue	nFeOOH-CG	
Cr	0.686	0.579	15
Pb	0.542	0.316	5
Cu	0.496	0.355	100
Cd	0.106	0.076	1
Zn	0.283	0.225	100
Ni	0.079	ND	5

### 3.6. nFeOOH-CG regeneration analysis

**Fig. 9:**
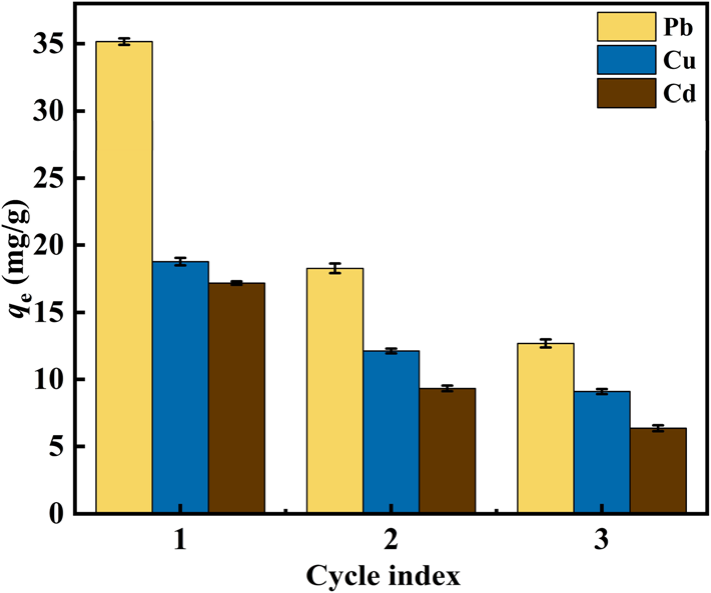
Recycling experiment of adsorption of Pb^2+^, Cu^2+^ and Cd^2+^ by nFeOOH-CG.

The regeneration and reuse performance of nFeOOH-CG were further studied by desorption and regeneration experiments to prove the practicability and economy of the adsorbent in actual wastewater treatment. As shown in Fig. 9, with the increase in the number of cycles, the removal rate of each metal ion decreased to varying degrees, indicating that the loss of some reducing substances may occur during the regeneration process, resulting in a decrease in FeOOH content and adsorption sites. After three adsorption–desorption cycles, the adsorption capacities of nFeOOH-CG for Pb^2+^, Cu^2+^ and Cd^2+^ were 12.86, 9.1 and 6.36 mg g^−1^, respectively, indicating that nFeOOH-CG had good regeneration performance.

### 3.7. Characterization analysis

#### 3.7.1. SEM-EDS analysis

**Fig. 10:**
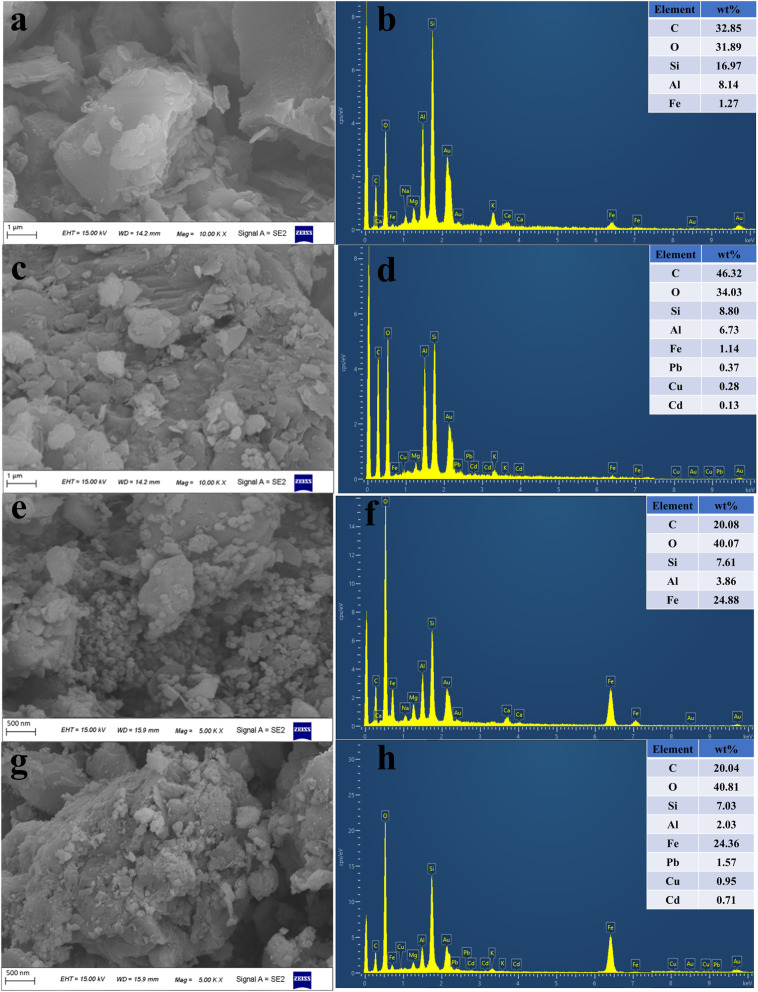
Comparative SEM-EDS characterization of coal gangue and nFeOOH-CG before and after treatment. (a) Before-treatment SEM image of coal gangue. (b) Before-treatment EDS of coal gangue. (c) After-treatment SEM image of coal gangue. (d) After-treatment EDS of coal gangue. (e) Before-treatment SEM image of nFeOOH-CG. (f) Before-treatment EDS of nFeOOH-CG. (g) After-treatment SEM image of nFeOOH-CG. (h) After-treatment EDS of nFeOOH-CG.

In order to understand the surface microstructure of coal gangue, nFeOOH-CG, the surface of the sample was observed by scanning electron microscopy, and the results are shown in Fig. 10. It can be seen from Fig. 10(a) and (c) that the surface of coal gangue is dense before treatment, showing an irregular block or lamellar structure. These porous characteristics provide more active sites for the adsorption of heavy metal ions. After the treatment, the surface of the coal gangue is rough, and a large number of folds and fine sediments appear. This is due to the long-term erosion of acidic wastewater, resulting in mineral dissolution and partial destruction of crystal structure.^47,48^ It can be seen from Fig. 10(b), (d) and Table 9 that lead, copper and cadmium elements appear after the treatment of coal gangue, and the mass percentages are 0.37%, 0.28% and 0.13%, respectively, indicating that the active sites are provided by the lamellar and porous structures on the surface of coal gangue^49,50^ so that metal ions such as Pb^2+^, Cu^2+^ and Cd^2+^ can be adsorbed on the surface or pores of coal gangue through intermolecular forces (van der Waals forces). It can be seen from Fig. 10(e) and (g) that before the treatment, the surface of nFeOOH-CG was loaded with well-dispersed nano-FeOOH spherical crystals, which were evenly distributed on the surface of coal gangue, which also increased the specific surface area and surface active sites of coal gangue. After the treatment, the spherical structure of nFeOOH-CG did not change significantly, indicating that the structure of α-FeOOH was relatively stable. It was observed that many small particles appeared on the surface of nFeOOH-CG. Combined with XRD analysis, Pb^2+^, Cu^2+^ and Cd^2+^ may be fixed on the surface of nFeOOH-CG in the form of complex state or low crystalline state. It can be seen from Fig. 10(f), (h) and Table 9 that there are obvious signals of lead, copper and cadmium on the surface of nFeOOH-CG after adsorption, and the mass percentages are 1.57%, 0.95% and 0.71%, respectively, which are much higher than that of single coal gangue. It indicated that nFeOOH-CG achieved efficient removal of Pb^2+^, Cu^2+^ and Cd^2+^ from AMD. The results further verify the feasibility and practicability of nFeOOH-CG as a treatment for heavy metals in AMD and also provide a strong basis for further study of the adsorption mechanism of nFeOOH-CG.

**Table 9:** Elemental composition of adsorbents before and after treatment

Type	Elemental composition (%)							
	C	O	Si	Al	Fe	Pb	Cu	Cd
Before-treatment coal gangue	32.85	31.89	16.97	8.14	1.27	0	0	0
After-treatment coal gangue	46.32	34.03	8.80	6.73	1.14	0.37	0.28	0.13
Before-treatment nFeOOH-CG	20.08	40.07	7.61	3.86	24.88	0	0	0
After-treatment nFeOOH-CG	20.04	40.81	7.03	2.03	24.36	1.57	0.95	0.71

#### 3.7.2. XRD analysis

**Fig. 11:**
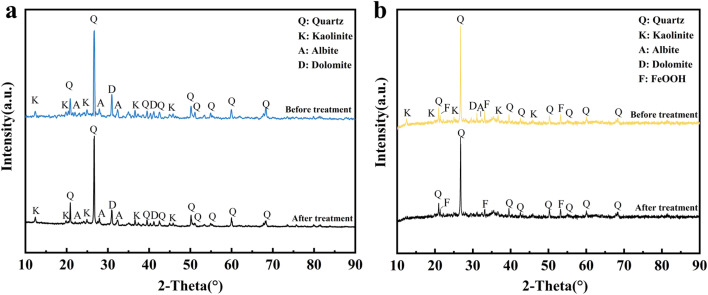
Comparative XRD patterns of coal gangue and nFeOOH-CG before and after treatment. (a) XRD patterns of coal gangue before and after treatment. (b) XRD patterns of nFeOOH-CG before and after treatment.

The X-ray diffraction patterns of Pb^2+^, Cu^2+^ and Cd^2+^ in AMD before and after treatment with coal gangue and nFeOOH-CG are shown in Fig. 11. It can be seen from Fig. 11(a) that the position of the characteristic diffraction peaks of kaolinite, quartz, albite and dolomite did not shift, but the intensity decreased compared with that after the adsorption reaction, indicating that the long-term erosion of acid wastewater (pH = 4) led to the dissolution of minerals (such as the carbonate component of dolomite) and the partial destruction of crystal structure, but did not change its basic phase composition.^51,52^ It can be seen from Fig. 11(b) that the characteristic diffraction peaks of α-FeOOH (PDF # 29-0713) appeared at 2*θ* values of 21.36°, 33.21° and 53.2°, indicating that α-FeOOH was successfully loaded. From the diffraction pattern of nFeOOH-CG reaction, it can be seen that the characteristic diffraction peak intensity of quartz is further weakened, and the characteristic diffraction peak of kaolinite disappears. However, the position of the characteristic diffraction peaks of α-FeOOH did not change and no new characteristic diffraction peaks appeared. However, the characteristic diffraction peaks at 2*θ* = 33.21° and 53.2° decreased after adsorption, indicating that the adsorption did not cause damage to the structure of α-FeOOH. Pb^2+^, Cu^2+^ and Cd^2+^ may be fixed on the surface of nFeOOH-CG in the form of complex state or low crystalline state.^53,54^

#### 3.7.3. FTIR analysis

**Fig. 12:**
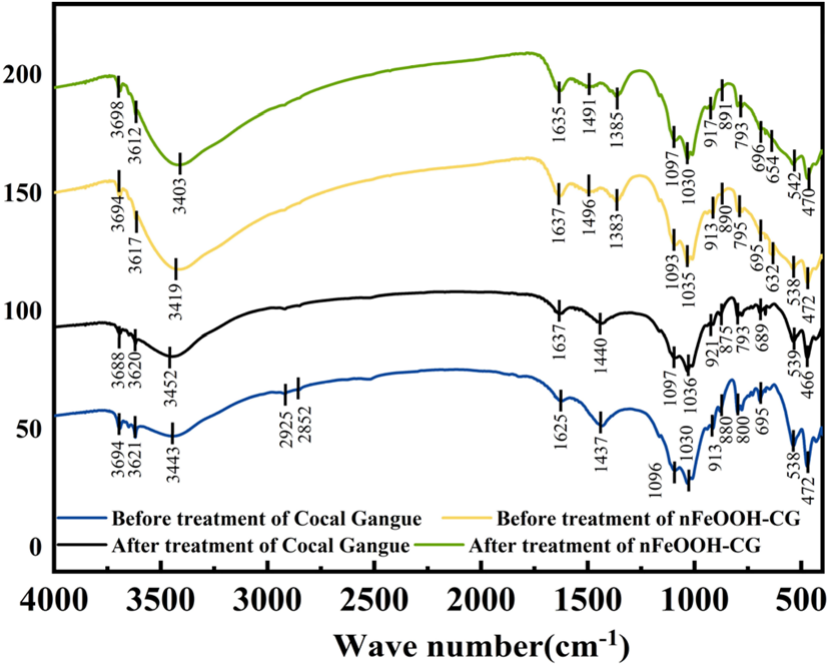
Comparative FTIR spectra of coal gangue and nFeOOH-CG before and after treatment.

The FTIR characterization results of Pb^2+^, Cu^2+^, and Cd^2+^ in AMD before and after treatment of coal gangue and nFeOOH-CG are shown in Fig. 12. From the infrared spectra of coal gangue before and after the reaction shown in Fig. 12, it can be seen that the infrared absorption peaks of coal gangue after the adsorption reaction are basically the same, but the intensity of the characteristic peaks has changed. The intensity of the –OH absorption peak at 3452 cm^−1^ is weakened, which is due to the destruction of the mineral skeleton (Si–O, Al–OH) of aluminosilicates such as kaolinite under acidic conditions. The H–O–H bending vibration peak at 1637 cm^−1^ is due to the vibration caused by the absorption of water by coal gangue.^55^ The Si–O–Si or Si–O–Al symmetric or antisymmetric stretching at 1000–1100 cm^−1^ is weakened, indicating that the crystal structure of some quartz is decomposed and destroyed. The above changes indicate that coal gangue mainly adsorbs heavy metal ions by physical adsorption, which is consistent with XRD and SEM analysis. The FTIR spectra of nFeOOH-CG before and after adsorption were compared. After the adsorption reaction, the –OH bond strength at 3419 cm^−1^ was significantly weakened and the peak position was significantly shifted, indicating that –OH on the surface of the composite material participated in the reaction, indicating that the surface of nFeOOH-CG may form an inner surface complex. At the same time, the band intensity of Fe–OH at 795 cm^−1^ and 890 cm^−1^ decreased, indicating that the oxygen atoms in –OH shared their non-bonded electron pairs with Pb^2+^, Cu^2+^ and Cd^2+^ and substituted H in the group to form Fe–O–Pb, Fe–O–Cu, and Fe–O–Cd surface complexes.^56^ Therefore, –OH of the nFeOOH-CG composite material is significantly increased compared with coal gangue, which increases the adsorption performance of heavy metal ions. In addition, the absorption peak of Fe–O at 632 cm^−1^ shifted to 654 cm^−1^ after the adsorption reaction, indicating that the Fe–O group in the nFeOOH-CG composite also participated in the reaction, indicating that Pb^2+^, Cu^2+^, and Cd^2+^ may replace part of Fe in α-FeOOH, which is consistent with the results of EDS analysis that the mass percentage of nFeOOH-CG before and after the reaction decreased from 24.88% to 24.36%. In summary, –OH, Fe–OH and Fe–O on the surface of nFeOOH-CG are involved in the chemical adsorption of heavy metal ions in AMD.

#### 3.7.4. BET analysis

**Fig. 13:**
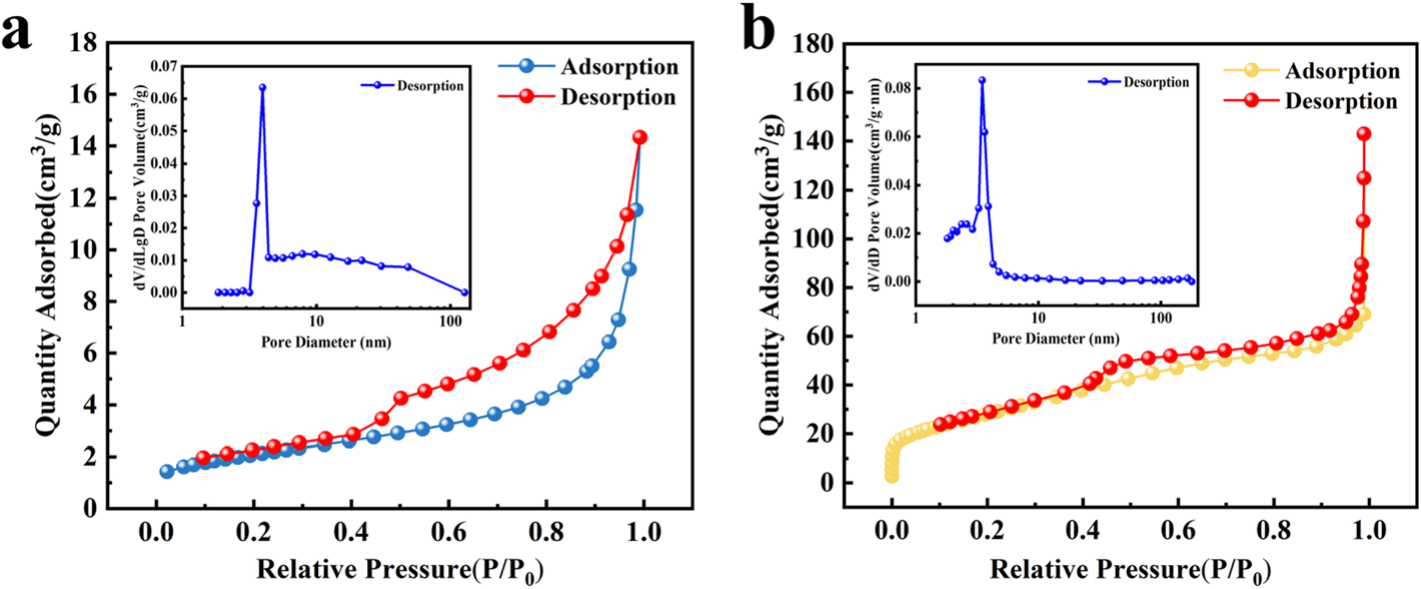
N_2_ adsorption–desorption isotherms and pore size distributions of coal gangue and nFeOOH-CG. (a) Coal gangue. (b) nFeOOH-CG.

According to the BET isothermal adsorption curve equation and the BJH method, the results are shown in Table 10. The N_2_ adsorption–desorption isotherms and pore size distribution curves of coal gangue and nFeOOH-CG are shown in Fig. 13. From Fig. 13(a), it can be seen that the adsorption–desorption isotherm curve of coal gangue is IV-type curve, accompanied by an H_3_-type hysteresis loop, indicating that the pores inside coal gangue are mostly slit-like mesopores or macropores. It can be seen from Fig. 13(b) that the N_2_ adsorption–desorption isotherms of nFeOOH-CG show type IV isotherm characteristics,^57^ which indicates that it has porosity, and capillary condensation occurs when the relative pressure increases, accompanied by a H_1_-type hysteresis loop, indicating that nFeOOH-CG is a typical mesoporous material. Table 10 shows the specific surface area, pore volume and pore size parameters of coal gangue and nFeOOH-CG. According to the calculation, the specific surface areas of coal gangue and nFeOOH-CG were 7.29 m^2^ g^−1^ and 103.68 m^2^ g^−1^, respectively. Compared with coal gangue, the specific surface area of nFeOOH-CG increased by 13.22 times, indicating that the introduction of nano-FeOOH greatly increased the specific surface area of the material. The pore volume of coal gangue and nFeOOH-CG were 0.021 cm^3^ g^−1^ and 0.221 cm^3^ g^−1^, respectively. The pore volume of nFeOOH-CG was significantly higher than that of coal gangue, indicating that the pore structure of nFeOOH-CG was more developed. The pore size of coal gangue is mainly concentrated at 11.51 nm, while the pore size of nFeOOH-CG is reduced to 8.52 nm, indicating that nano-FeOOH particles are loaded on the surface or pores of coal gangue, which may fill some of the original mesopores or macropores, resulting in a decrease in the overall pore size. The pore size distribution of nFeOOH-CG shows that the pore size of nFeOOH-CG is mainly concentrated in 3–10 nm. Obviously, the high specific surface area of nFeOOH-CG is the key to remove Pb^2+^, Cu^2+^ and Cd^2+^ pollutants in AMD.

**Table 10:** N_2_ adsorption–desorption isotherm curves and pore size distribution curves of coal gangue and nFeOOH-CG

Name	Specific surface area (m^2^ g^−1^)	Pore volume (cm^3^ g^−1^)	Pore size (nm)
Coal gangue	7.29	0.021	11.51
nFeOOH-CG	103.68	0.221	8.52

### 3.8. Adsorption mechanism

**Fig. 14:**
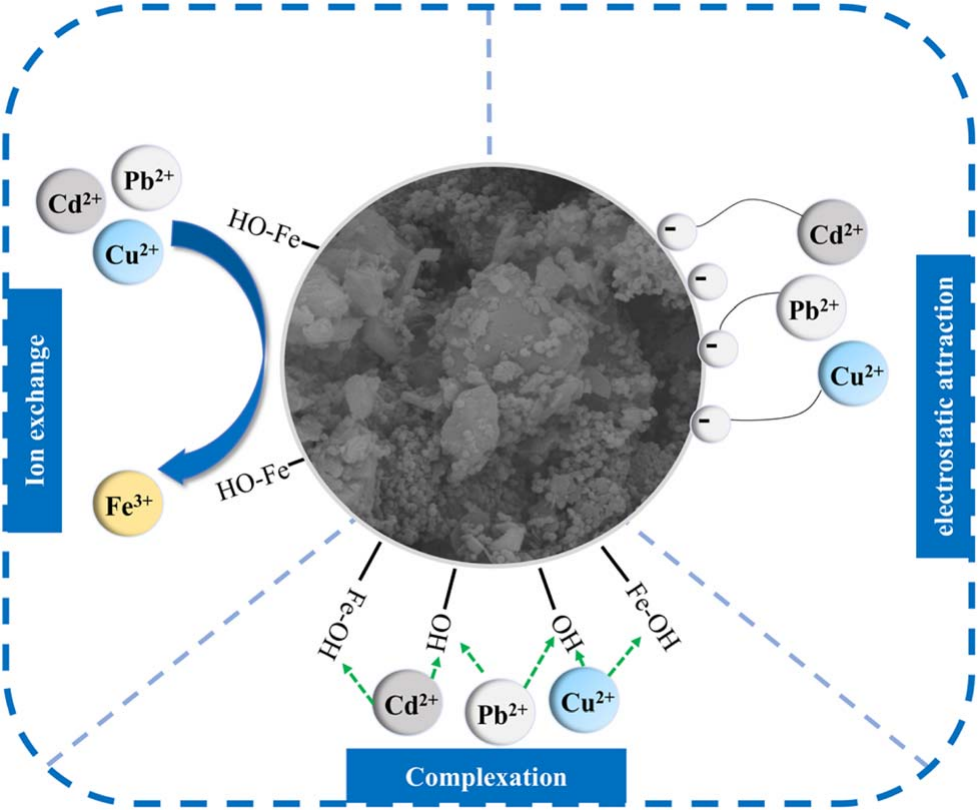
Proposed adsorption mechanism of nFeOOH-CG for heavy metal removal from AMD.

Through performance evaluation experiments investigating the effects of various factors, studies on adsorption isotherms, adsorption kinetics, and adsorption thermodynamics, and the characterization of nFeOOH-CG, the adsorption mechanism of nFeOOH-CG for Pb^2+^, Cu^2+^ and Cd^2+^ in AMD was elucidated. The results demonstrated that nFeOOH-CG exhibits exceptional adsorption performance for heavy metal ions in AMD environments. This can be explained from two aspects: structural characteristics and surface activity. In terms of structural characteristics, the SEM characterization shows that the surface of coal gangue is obviously covered with a layer of uniformly distributed spherical nano-FeOOH particles after loading modification, the pore structure is formed between the grains, and the pore structure is developed. BET characterization showed that the specific surface area and pore volume of nFeOOH-CG increased significantly, providing more adsorption sites. The excellent pore structure is conducive to the diffusion of Pb^2+^, Cu^2+^ and Cd^2+^ into the pores. FTIR characterization showed that the intensity of the-OH stretching vibration peak of FeOOH decreased and the Fe–O characteristic peak shifted after adsorption, indicating that the heavy metal had a coordination reaction with the surface hydroxyl group. BET characterization showed that the specific surface area of coal gangue increased significantly after loading nano-FeOOH, and the developed pore structure promoted ion diffusion. EDS surface scanning confirmed that Pb^2+^, Cu^2+^ and Cd^2+^ were specifically enriched in the FeOOH nanoparticle area. This is consistent with the results of BET analysis. In addition, the interaction between nFeOOH-CG and Pb^2+^, Cu^2+^, and Cd^2+^ is also crucial for high adsorption capacity. In terms of surface activity, according to the adsorption isotherm and adsorption kinetics, the adsorption of Pb^2+^, Cu^2+^ and Cd^2+^ in AMD by nFeOOH-CG conforms to the Langmuir model (*R*^2^ = 0.99312, 0.99967, 0.99797) and the pseudo-second-order kinetic model (*R*^2^ = 0.99962, 0.99871, 0.99934), indicating that the adsorption process is monolayer adsorption, mainly chemical adsorption. This is mainly because the –OH on the surface of nFeOOH-CG participates in the reaction. The oxygen atoms in the –OH share their non-bonded electron pairs with Pb^2+^, Cu^2+^, and Cd^2+^ and replace the H in the group to form Fe–O–Pb, Fe–O–Cu, and Fe–O–Cd surface complexes. Moreover, the Fe–O group in nFeOOH-CG composites participated in the reaction, and some Pb^2+^, Cu^2+^ and Cd^2+^ may replace Fe in α-FeOOH.^58^ According to the study of adsorption thermodynamics, the adsorption of Pb^2+^ and Cu^2+^ by nFeOOH-CG is a spontaneous, endothermic and entropy-increasing process, while the adsorption of Cd^2+^ is a non-spontaneous, endothermic and entropy-increasing process. In addition, the initial pH of the solution will affect the physical and chemical adsorption reactions such as electrostatic interaction, complexation reaction and ion exchange between the pollutant and the surface of the adsorption material. This is because in the lower pH range (pH = 2–3), the surface hydroxyl of the supported nano-FeOOH protonates to form ≡FeOH^2+^, which is mutually exclusive with Pb^2+^, Cu^2+^, and Cd^2+^. The removal rate of Pb^2+^, Cu^2+^, and Cd^2+^ by nFeOOH-CG is low. With the increase in pH, the concentration of OH^−^in the solution is high, which will increase the negative charge on the surface of nFeOOH-CG and reduce the electrostatic repulsion with Pb^2+^, Cu^2+^ and Cd^2+^, which is beneficial to the adsorption or formation of insoluble precipitates and improves the removal effect. This also shows that the adsorption of adsorbates by nFeOOH-CG is related to the electrostatic interaction between the two. In summary, the adsorption of Pb^2+^, Cu^2+^ and Cd^2+^ in AMD by nFeOOH-CG mainly depends on complexation, ion exchange and electrostatic attraction. In summary, the adsorption of Pb^2+^, Cu^2+^ and Cd^2+^ in AMD by nFeOOH-CG achieved efficient adsorption through the synergistic effect of complexation, ion exchange, and electrostatic attraction. This multi-mechanism model has been cross-validated by surface analysis techniques and adsorption models. The schematic diagram of the adsorption mechanism is shown in Fig. 14.

## 4 Conclusions

In this study, based on the stable mineral skeleton structure of coal gangue and the good adsorption of nano-FeOOH, a new adsorbent, coal gangue-supported nano-FeOOH composite, was prepared by mineral loading technology and chemical precipitation method. The effects of various factors on the removal of Pb^2+^, Cu^2+^ and Cd^2+^ by nFeOOH-CG were investigated by a performance evaluation test. The results showed that under the conditions of dosage of 5 g L^−1^, initial pH of 4, contact time of 150 min and initial concentration of 100 mg L^−1^, the removal rates of Pb^2+^, Cu^2+^ and Cd^2+^ in AMD by nFeOOH-CG were 96.85%, 88.38% and 73.1%, respectively, which were significantly higher than those of coal gangue. The adsorption of Pb^2+^, Cu^2+^ and Cd^2+^ in AMD by nFeOOH-CG conforms to the Langmuir model (*R*^2^ = 0.99312, 0.99967, and 0.99797) and the pseudo-second-order kinetic model (*R*^2^ = 0.99962, 0.99871, and 0.99934), indicating that the adsorption process is monolayer adsorption and dominated by chemical adsorption. The adsorption mechanism of Pb^2+^, Cu^2+^ and Cd^2+^ in AMD by nFeOOH-CG is attributed to the increased specific surface area, complexation, ion exchange and electrostatic attraction. The nFeOOH-CG can maintain efficient adsorption performance under different pH and different concentrations of Pb^2+^, Cu^2+^, and Cd^2+^, showing a wide range of application potential. In summary, the nFeOOH-CG prepared with coal gangue as the load carrier can be simply and effectively used as an adsorbent to remove Pb^2+^, Cu^2+^ and Cd^2+^ from AMD in an environmentally friendly manner. The nFeOOH-CG is expected to be applied in industry on a large scale, and this study provides new ideas and technical support for the high-value utilization of coal gangue.

## Author contributions

Xuying Guo: conceptualization, methodology, validation, formal analysis, and writing – original draft preparation. Xiaoyue Zhang: software, resources, data curation, visualization, and writing – review and editing. Zilong Zhao: resources, project administration, and supervision. Yanrong Dong: project administration, supervision, and validation. Honglei Fu: conceptualization, investigation, and writing – original draft preparation. Wei Sun: investigation, project administration, and supervision. Fanbo Meng: conceptualization, methodology, resources, and supervision.

## Conflicts of interest

The authors declare that they have no known competing financial interests or personal relationships that could have appeared to influence the work reported in this paper.

## Data Availability

Data will be made available upon request.
